# Yeast Culture Supplementation Effects on Systemic and Polymorphonuclear Leukocytes’ mRNA Biomarkers of Inflammation and Liver Function in Peripartal Dairy Cows †

**DOI:** 10.3390/ani13020301

**Published:** 2023-01-15

**Authors:** Nathaly Ana Carpinelli, Jessica Halfen, Tainara Cristina Michelotti, Fernanda Rosa, Erminio Trevisi, Jim D. Chapman, Evin S. Sharman, Johan S. Osorio

**Affiliations:** 1 Department of Dairy and Food Sciences, South Dakota State University, Brookings, SD 57007, USA; 2 Nucleo de Pesquisa, Ensino e Extensao em Pecuaria, Universidade Federal de Pelotas, Pelotas 96010610, Rio Grande do Sul, Brazil; 3 Department of Animal Sciences, Food and Nutrition (DIANA), Facoltà di Scienze Agrarie, Alimentari e Ambientali, Università Cattolica del Sacro Cuore, 29122 Piacenza, Italy; 4 Phibro Animal Health, Teaneck, NJ 07666, USA

**Keywords:** transition period, inflammation, yeast culture, immune response

## Abstract

**Simple Summary:**

During the transition period (3 weeks pre- to 3 weeks postpartum), dairy cows experience metabolic and immune alteration that can lead to a higher incidence of health disorders during early lactation. Therefore, we proposed the supplementation with yeast-based products in dairy cows’ diets during the transition period as a nutritional strategy to enhance the immune responses during this challenging period. Our results suggest that yeast culture could stimulate a more active inflammatory response, with signs of a resolution of the inflammation in transition cows; however, the specific mechanism of how bioactive compounds in yeast culture elicit this response during the transition period remains unknown.

**Abstract:**

This study evaluated the effects of feeding a commercial yeast culture on blood biomarkers and polymorphonuclear leukocyte (PMNL) gene expression in dairy cows during the transition period until 50 d postpartum. Forty Holstein dairy cows were used in a randomized complete block design from −30 to 50 d. At −30 d, cows were assigned to a basal diet plus 114 g/d of top-dressed ground corn (control; *n* = 20) or 100 g/d of ground corn and 14 g/d of a yeast culture product (YC; *n* = 20). Blood samples were collected at various time points from −30 to 30 DIM to evaluate blood biomarkers and PMNL gene expression related to inflammation, liver function, and immune response. Liver function biomarkers, gamma-glutamyl transferase (GGT) and albumin were greater and lower, respectively, in YC cows in comparison to control. However, these biomarkers remained within physiological levels, indicating an active inflammatory process. Genes in PMNL expression related to inflammation (*NFKB1*, *TNFA*, *TRAF6*), anti-inflammation (*IL10*), and cell membrane receptors (*SELL*) were upregulated in the YC group in comparison to control. These results suggest that YC could stimulate a more active inflammatory response with signs of a resolution of inflammation in transition cows.

## 1. Introduction

The transition period encompasses the period from 3 weeks before through 3 weeks after parturition [1], and it is the most critical period in the lactation cycle of dairy cows. The metabolic stress and altered immune function may contribute to the noticeable incidence of health disorders during early lactation [2,3]. The development of nutrition-based strategies that can modulate the immune response has the potential to positively affect dairy cows’ health. Yeast-based products have been used in livestock for many years due to their beneficial effects on animals, with the most common species being *Saccharomyces cerevisiae*.

Yeast-based product supplementation during the transition period has been shown to improve DMI [4,5] and milk yield [6,7], while the same effect was not observed by others [8]. Recent findings also suggest that yeast may influence immune function. However, the mechanism behind the interaction between supplemented yeast and innate immunity is still unclear. According to Lopreiato et al. [9], the responses can be attributed to a better energy status promoted by positive effects on digestive function or activation of the immune system by sensing yeast components.

Jensen et al. [10] demonstrate that yeast culture provides antioxidant, anti-inflammatory, and immunomodulatory activities in vitro. Moreover, Yuan et al. [11] reported that yeast culture plus enzymatically hydrolyzed yeast could enhance humoral and mucosal immunity, modulate uterine inflammatory signals, and regulate mammary gland health in transition dairy cows. Recently, Knoblock et al. [12] evaluated the effects of a *Saccharomyces cerevisiae* fermentation product in dairy cows fed diets with different starch content during the early lactation and observed that yeast supplementation coupled with low-starch diets might reduce inflammation. In lactating dairy cows, yeast cell wall supplementation has been associated with increased mRNA expression of cytokines in peripheral blood mononuclear cells, underscoring yeast’s ability to mediate immune function [13]. Therefore, we hypothesized that supplementing a yeast culture fermentation product around parturition would modulate the immune response and antioxidant systems coupled with positive health and performance in transition dairy cows. The objective of this study was to assess the effects of a yeast culture product on metabolism, inflammation, liver function, and immune function through blood biomarkers and gene expression in polymorphonuclear leukocytes (PMNLs) in dairy cows during the transition period until 50 DIM.

## 2. Materials and Methods

### 2.1. Animals, Experimental Design, and Dietary Treatments 

The Institutional Animal Care and Use Committee (IACUC) of South Dakota State University approved all the procedures for this study (protocol no. 18-028A). Details of the experimental design have been published previously [14]. Briefly, 43 Holstein dairy cows, including 35 multiparous and 8 primiparous cows, were used in a randomized complete block design and were blocked according to expected calving day, parity, and previous lactation milk yield (11,498 ± 2328 kg) for multiparous cows or genetic merit for primiparous. A total of 3 cows were removed from the experiment due to calving with <14 d on treatment diets (*n* = 2) and twin calving (*n* = 1). Cows received the same basal diet once a day (1.39 Mcal of NE_L_/kg and 12.3% CP) from −30 d to parturition. After calving, all cows received the same basal lactation diet once a day (1.60 Mcal of NE_L_/kg and 15.6% CP). Both diets were fed as a total mixed ration. At −30 d before calving, cows were assigned to either a basal diet (control; 114 g/h/d ground corn; *n* = 20) or basal plus the Culture Classic HD yeast culture (YC; 100 g ground corn + 14 g of YC/d; *n* = 20). The treatments were top-dressed once a day from −31 ± 6 to 50 DIM.

The cows were enrolled in the experiment from mid-September 2018 to early April 2019. Cows were fed using an individual gate system (American Calan, Northwood, NH, USA), and the intake of dry matter (DM) was recorded daily. Diets were formulated using the CNCPS model (v. 6.55) [15] contained in the NDS professional ration formulation software (v. 6.55; RUM&N, NDS Professional, Reggio Emilia, Italy). Dry matter content of individual feed ingredients was determined weekly, and diets were adjusted accordingly to maintain formulated DM ratios of ingredients in the TMR. Cows were fed once daily at 0500 h before morning milking, and feed offered was adjusted daily to achieve 5 to 10% refusals.

During the dry period, the cows were housed in a bedded-pack pen, and 3 d before expected calving date, the cows were reallocated in individual pens bedded with straw until parturition. On d 3 postpartum, cows were moved to a lactation free-stall barn where they received the same lactation basal diet until 50 DIM. Body weight was measured weekly for each cow at the same time in the afternoon at 1300 h. Body condition score (BCS) (scale 1 = thin to 5 = obese) was assigned by 2 individuals, and the average score was used for statistical analysis. 

### 2.2. Blood Collection and Analyses

Blood was sampled from the coccygeal vein before morning feeding using a 20-gauge BD Vacutainer needle (Becton Dickinson, Franklin Lakes, NJ, USA) at −30, −15, 7, 14, and 30 d relative to parturition from a subset of multiparous cows (*n* = 8 cows/treatment). Blood was collected into the Vacutainer (5 mL, BD Vacutainer, Becton Dickinson, Franklin Lakes, NJ, USA) containing either serum clot activator or lithium heparin to obtain serum or plasma, respectively. After collection, tubes containing lithium heparin were placed on ice, and tubes with serum clot activator were kept at 21 °C until centrifugation. Serum and plasma were obtained by centrifugation at 1300× *g* for 15 min at 21 °C and 4 °C, respectively. Aliquots were frozen at −80 °C until further analysis. 

Samples were analyzed for biomarkers of liver function (e.g., total bilirubin, aspartate aminotransferase (AST/GOT), γ-glutamyl transpeptidase (GGT), cholesterol, and paraoxonase (PON)) and inflammation (e.g., albumin, ceruloplasmin, haptoglobin, and interleukin 1β (IL-1β)], using kits purchased from Werfen—Instrumentation Laboratory (Lexington, MA), and the procedures were described by [16,17,18].

### 2.3. PMNL Isolation

PMNLs were isolated based on procedures described by Osorio et al. [19]. Briefly, blood (~100 mL) was collected into evacuated tubes (8 mL, BD Vacutainer, Becton Dickinson, Franklin Lakes, NJ) containing solution A of trisodium citrate citric acid and dextrose (ACD) from the coccygeal vein prior to morning feeding at −30, 5, 15, and 30 days relative to parturition from a subset of multiparous cows (*n* = 8 cows/treatment). The isolation protocol consisted of lysing red blood cells with ice-cold deionized water, cleaning remaining PMNLs with 1× PBS, and restoring the iso-osmotic environment with 5× PBS. The final PMNL pellet was homogenized in 1 mL of Trizol reagent (Invitrogen, Carlsbad, CA, USA) and stored at −80 °C until further RNA isolation and gene expression analysis. 

### 2.4. RNA Isolation, cDNA Synthesis, and Real-Time Quantitative PCR

Total RNA was extracted from blood PMNLs using Trizol (Invitrogen, Carlsbad, CA, USA) reagent in combination with the RNeasy^®^ Plus Mini Kit (Qiagen, Hilden, Germany), following the manufacturer’s instructions with some modifications. Briefly, the cell pellet immersed in Trizol was transferred to a 2 mL RNase-free O-ring tube, containing one 5 mm stainless steel bead (Qiagen, Hilden, Germany) and homogenized in a Beadbeater (BioSpec Products, Bartlesville, OK, USA) for 1 min. After homogenization, the lysate was transferred to a 2 mL RNase-free microtube, and 200 µL of phenol: chloroform (Invitrogen, Carlsbad, CA, USA) at 4 °C was added in order to isolate the RNA from the organic phase. After centrifugation at 13,000× *g* for 15 min at 4 °C, the upper phase was transferred into a new 2 mL RNase-free microtube. The RNA quantity for all PMNL samples was 327.17 ± 292.99 ng/µL, and the purity (260/280) was 2.02 ± 0.10; these were determined using a NanoDrop spectrophotometer (ND 1000, NanoDrop Technologies Inc., Wilmington, DE, USA). The RNA integrity was assessed using the TapeStation (Agilent Technologies, Santa Clara, CA, USA), and the final RNA integrity number was 5.6 ± 1.7. 

RNA isolated from the PMNLs was used for qPCR analysis. Complementary DNA (cDNA) synthesis was performed according to Osorio et al. [20] with modifications. cDNA was synthesized using 100 ng RNA, 1 µL random primers (Invitrogen Corp., Waltham, MA, USA), and 9 µL DNase/RNase-free water (HyClone, UltraPure™, Cytiva, Malborough, MA, USA). The mixture was incubated at 65 °C for 5 min and kept on ice for 5 min. A total of 9 µL of master mix composed of 4 µL 5× Reaction Buffer, 1 µg dT18 (Operon Biotechnologies, Huntsville, AL, USA), 2 µL 10 mmol/L dNTP mix (Invitrogen Corp.), 0.25 µL (50 U) of RevertAidTM Reverse Transcriptase (Fermentas Inc., Waltham, MA, USA), 0.125 µL of RNase Inhibitor (10 U, Promega, Madison, WI, USA), and 1.625 µL of DNase/RNase free water (HyClone, UltraPure™) was added. The reaction was performed in an Eppendorf Mastercycler^®^ Gradient using the following temperature program: 25 °C for 5 min, 42 °C for 120 min, and 70 °C for 5 min. The real-time quantitative PCR was performed according Rosa et al. [21] using a QuantStudio™ 6 Flex Real-Time PCR System (Applied Biosystems, Walthan, MA, USA) following the conditions: 2 min at 50 °C, 10 min at 95 °C, 40 cycles of 15 s at 95 °C (denaturation), and 1 min at 60 °C (annealing + extension). 

Details about the primer sequence information and sources can be found in Supplementary Table S1. In this study, the genes Golgin A5 (*GOLGA5*), Oxysterol Binding Protein Like 2 (*OSBPL2),* and Single-Strand-Selective Monofunctional Uracil-DNA Glycosylase 1 (*SMUG1*) were used as internal control genes (ICGs), as described by Moyes et al. [22]. The final gene expression data were normalized with the geometric mean of the 3 internal control genes. The stability of the ICG was assessed using the geNorm software [23] with a favorable final pairwise variation of 0.17.

Target genes measured are described in Supplemental Table S2, and these were related to inflammation (tumor necrosis factor alpha (*TNFA*), nuclear factor of kappa light polypeptide gene enhancer in B-cells 1 (*NFKB1*), interleukin 1B (*IL1B*) and interleukin 10 (*IL10*), TNF receptor associated factor 6 (*TRAF6*), and myeloid differentiation primary response 88 (*MYD88*)), cell receptors and signaling (toll-like receptor 2 (*TLR2*), toll-like receptor 4 (*TLR4*), selectin-L (SELL), interleukin 1 receptor associated kinase 1 (*IRAK1*), Z-DNA binding protein 1 (*ZBP1*), integrin subunit alpha M (*ITGAM*), talin 1 (*TLN1*), and vinculin (*VCL*)), and oxidative stress (nuclear factor erythroid 2-related factor 2 (*NRF2*), superoxide dismutase 1 (*SOD1*), superoxide dismutase 2 (*SOD2*), and myeloperoxidase (*MPO*)).

### 2.5. Statistical Analysis

Blood biomarkers and gene expression data were analyzed using the MIXED procedure of SAS 9.4 (SAS Institute Inc., Cary, NC, USA) using treatment, time (day or week), parity, and 2- and 3-way interactions with treatment as fixed effects and cow nested within treatment as a random effect. Parity and its interactions were tested and removed from the model when *p* > 0.20.

The exponential correlation covariance structure SP for repeated measures was used for the analysis of gene expression and blood biomarker data with the following model:

$$y_{ijk\;}=\mu +D_{i}+P_{j}+DP_{ij}+T_{k}+DT_{ik}+DPT_{ijk}+\alpha_{l}+e_{ijkl}\;$$

where *y_ijk_* is the dependent, continuous variable; *μ* is the general mean; *D*_i_ is the fixed effect of the treatment (*i* = 1 or 2, namely, control or YC); *P_j_* is the fixed effect of the *j*th parity (*j* = 2nd or 3rd), DP*_ij_* is the fixed effect of the *i*th treatment by the *j*th parity of the experiment interaction; T*_k_* is the fixed effect of the *k*th time (*j* = 1, 2, 3, or 4, namely, −30, 5, 15, and 30 DIM); *DT_ik_* is the fixed effect of the *i*th treatment by the *k*th time of the experiment interaction; *DPTijk* is the fixed effect of the *i*th treatment by the *j*th parity by the *k*th time of the experiment interaction; α*_l_* is the random effect of individual; *e_ijkl_* is the residual error.

Blood biomarker data were log-scale transformed if needed to comply with normal distribution of residuals and subsequently back-transformed. Gene expression data were transformed as fold change relative to −30 d and then log-transformed prior to statistical analysis. Data on day −30 were used as a covariate for blood biomarkers. Data were analyzed as repeated measures using the exponential correlation covariance structure (POW). The covariate of previous lactation milk yield was maintained in the model for all variables for which it was significant (*p* < 0.05). Separation of least square means for significant effects was accomplished using the Tukey option within the MIXED procedure of SAS. Statistical significance was declared at *p* ≤ 0.05 and tendencies at *p* < 0.10. 

## 3. Results

### 3.1. Blood Biomarkers 

**Figure 1:**
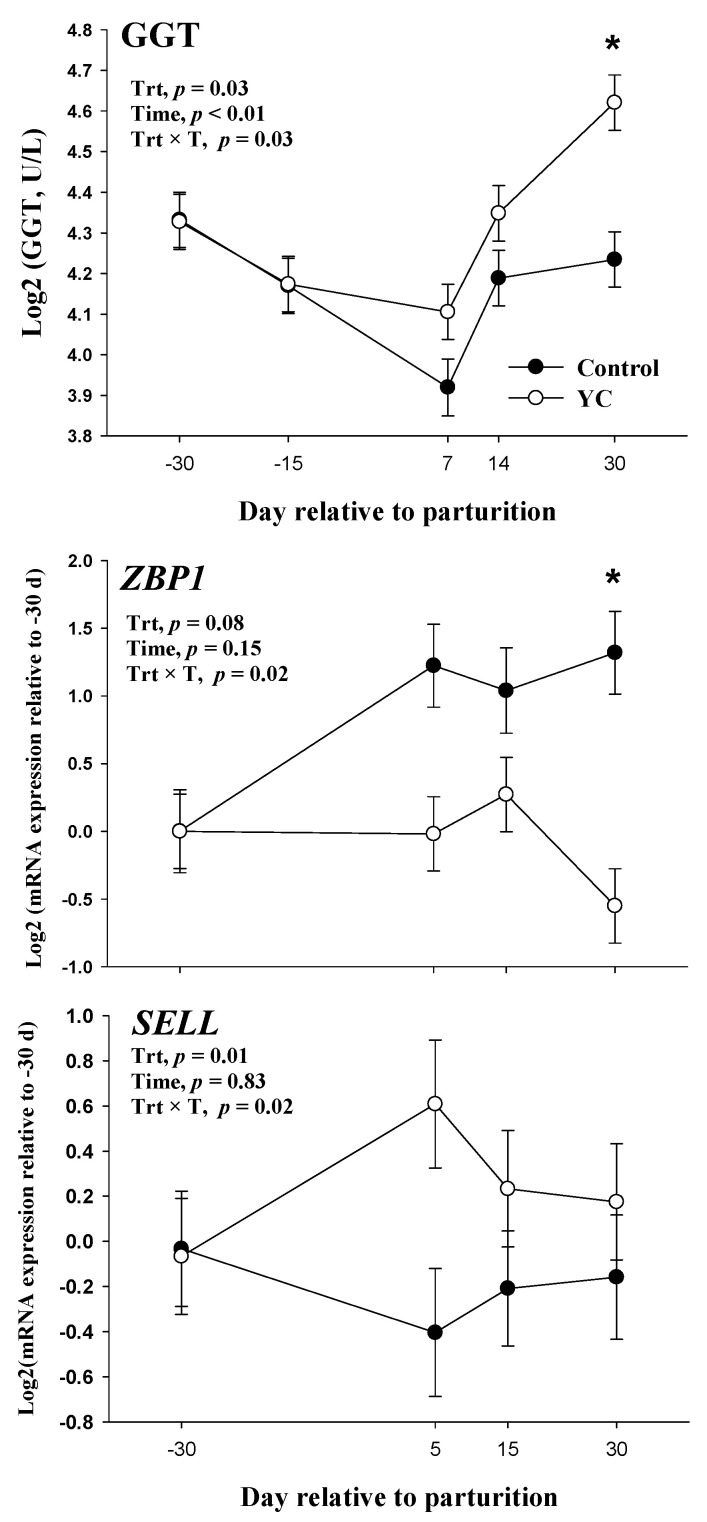
Blood gamma-glutamyl transferase (GGT) and gene expression of Z-DNA binding protein 1 (*ZBP1*) and L-selectin (*SELL*) in polymorphonuclear leukocytes in cows (*n* = 8/treatment) supplemented with a basal diet (Control) or a basal diet plus yeast culture fermentation production (YC) from −31 ± 6 d relative to parturition through 50 DIM. Mean separations between treatments at a given time point were evaluated when a treatment × time (Trt × T) interaction (*p* ≤ 0.05) was observed, and differences (*) were declared at a Tukey-adjusted *p* ≤ 0.05. Values are means, and SE are represented by vertical bars.

Main effects of diet, time, and their interactions for blood metabolites associated with liver function and inflammation are presented in Table 1. There was a treatment × time (Trt × T) interaction (*p* = 0.03) in GGT (Figure 1), which resulted in greater (*p* < 0.01) concentrations of GGT in YC-fed cows than control at 30 DIM. This effect was reflected in an overall greater (*p* = 0.03) GGT in YC cows. In contrast to GGT, the concentration of albumin was lower (*p* < 0.01) in YC-fed cows when compared to control. Total bilirubin, GOT, cholesterol, PON, ceruloplasmin, haptoglobin, and IL-1β were not affected by treatment, time, or their interaction.

Among the blood biomarkers evaluated, only GOT had a Parity × Trt × T interaction (*p* = 0.05). However, this effect was not associated with differences across parity and treatments at any given time point. Similarly, a Parity × T interaction (*p* = 0.05) observed in PON did not result in differences between second and third lactation cows at any time point evaluated. Unlike PON, the Parity × T in GGT was reflected in greater (*p* = 0.04) GGT in third lactation cows than second lactation at 30 DIM.

### 3.2. Gene Expression 

Main effects of diet, time, and their interactions for PMNL mRNA gene expression are present in Table 2.

#### 3.2.1. Inflammation

The mRNA expression of *TNFA*, *NFKB1*, and *IL10* was greater (*p* = 0.04) in YC when compared to control cows. There was a tendency (*p* = 0.07) of an upregulation of *TRAF6* in cows supplemented with YC compared to control. Additionally, a Parity × Trt interaction (*p* = 0.04) was observed for *IL10*, in which second lactation YC-fed cows had a greater (*p* = 0.01) mRNA expression than second lactation control cows (Supplementary Figure S1). 

#### 3.2.2. Cell Receptors and Signaling 

There was a Trt × T interaction (*p* = 0.02) in *ZBP1* and *SELL* (Figure 1), which resulted in greater (*p* < 0.01) *ZBP1* mRNA expression in YC-fed cows at 30 DIM. There was an evident upregulation of *SELL* in YC-fed cows in comparison to control at 5 DIM (Figure 1) that could have explained the Trt × T in *SELL*; however, this effect did not reach statistical differences (*p* = 0.23). These effects were reflected in overall greater (*p* ≤ 0.01) mRNA expression of *SELL* and *ZBP1* in cows supplemented with YC in comparison to control. Additionally, there was a tendency (*p* = 0.08) of an upregulation of *VCL* in cows supplemented with YC compared to control.

We observed a Parity × Trt × T interaction (*p* = 0.02) in *ZBP1*, in which second lactation YC-fed cows had a greater mRNA expression at 5, 15, and 30 DIM when compared to second lactation control cows. This effect resulted in an overall Parity × Trt interaction (*p* < 0.01), where second lactation YC-fed cows had a greater mRNA expression of *ZBP1* than second lactation control cows; however, no differences were observed for third lactation cows (Figure S1). A Parity × Trt interaction (*p* = 0.02) was also observed for *VCL,* where second lactation YC-fed cows had a greater (*p* = 0.01) mRNA expression than second lactation control cows (Figure S1).

#### 3.2.3. Oxidative Stress

There was a tendency (*p ≤* 0.09) of an upregulation of *NRF2* and *SOD1* in cows supplemented with YC in comparison to control. In contrast, we observed a trend (*p* = 0.10) for the downregulation of *MPO* mRNA expression in YC-fed cows when compared to control cows. Similar to *ZBP1*, *VCL*, and *IL10*, there was a Parity × Trt interaction (*p* = 0.05) for *SOD1* mRNA expression, where SOD1 was upregulated (*p* = 0.02) in second lactation YC-fed cows in comparison to second lactation control cows, while no differences were observed between treatments in third lactation cows (Figure S1).

## 4. Discussion

Effects on performance parameters, blood biomarkers (i.e., muscle body mass, metabolism, and oxidative stress), rumen fermentation profile, and rumen bacterial abundance for this study have been published in a companion manuscript [14]. In summary, we observed a trend for a diet effect in energy-corrected milk (ECM), where YC cows (38.4 ± 1.2 kg/d) produced 3.2 kg/d more milk than control cows (35.2 ± 1.2 kg/d). Yeast culture supplementation also promoted alterations in rumen fermentation by increasing valerate soon after calving with a concomitant increase in rumen microbiota populations of cellulolytic and lactic acid-utilizing bacteria. The current manuscript focused on systemic inflammation and PMNL mRNA biomarkers in response to yeast supplementation during the transition period.

### 4.1. Inflammation

During the transition period, dairy cows are more susceptible to health disorders such as mastitis, ketosis, and retained placenta. A common factor associated with the development of these disorders is immune dysfunction [24], which, in turn, is caused by metabolic changes during the transition period. During inflammation, pro-inflammatory cytokines elicit a systemic effect by activating leukocytes and endothelial cells [25] while stimulating hepatocytes to increase or decrease the synthesis of acute-phase proteins (APP). These proteins can be further classified as positive or negative APP if their blood concentration increases or decreases during inflammation. Alterations in acute-phase proteins have been used to monitor dairy cows’ health, especially during the transition from pregnancy to lactation [18,26]. 

Albumin is an important negative APP in ruminants, and decreased levels are correlated with an inflammatory process [27]. Moreover, albumin is commonly used as a biomarker of liver function around calving [28]. In a retrospective analysis, Bertoni et al. [29] classified early lactation dairy cows based on their liver function through a liver activity index (LAI). They evaluated the impact of LAI on APP concentrations and lactation performance. The albumin levels at 7 DIM from cows classified as high- and low-LAI were 34.5 g/L and 29.0 g/L, respectively. In the present study, albumin reached nadir levels at 7 DIM, regardless of treatments (Supplementary Figure S2), at which point concentrations were 35.2 and 33.3 g/L in control and YC cows, respectively. This suggests that YC cows, even lower than control, remained at comparable levels to cows with a high LAI in Bertoni et al. [29]. Moreover, high-LAI cows in Bertoni et al. [29] maintained a greater milk production than low-LAI cows during the first 4 wk postpartum (36.1 vs. 29.5). This further strengthens our hypothesis that while YC cows had lower albumin concentration than control, this effect was not consistent with a detrimental inflammation, which was also supported by the trend (*p* = 0.07) for greater ECM in YC cows than control (38.4 vs. 35.2) [14].

The effect of yeast supplementation on albumin concentrations has been reported by different studies but with inconsistent results. Mumbach et al. [28] observed similar serum albumin (24.7 vs. 24.8 ± 0.06) between yeast-culture-supplemented cows and control during early postpartum (from calving to 15 ± 3 DIM); however, lower albumin was observed by early lactation (15 to 18 ± 3 DIM) in yeast-culture-supplemented cows in comparison to control cows (23.1 vs. 25.6 ± 0.08). Furthermore, when compared to control cows, Faccio-Demarco et al. [30] observed that transition cows supplemented with yeast culture had lower albumin levels at 3 and 7 DIM; however, this effect did not translate into an overall treatment effect. 

### 4.2. Liver Function

Although not specific to the liver, the enzyme GGT is a marker of liver cell damage, and high GGT levels have been associated with cell necrosis [29,31]. In the current study, we observed an increase in GGT in YC cows, and this effect was more pronounced at 30 DIM, where GGT levels for YC cows were 24.6 U/L compared to 18.8 U/L for control cows. The latter effect corresponded to a 25.8% increase in GGT from −30 DIM to 30 DIM (Figure S2). However, these GGT levels in YC cows were comparable to those observed in the high-LAI group in Bertoni et al. [29]. In fact, GGT was not different across LAI-based groups, suggesting that the LAI parameter does not account for liver cell damage [29]. Moreover, according to Bertoni et al. [29], impaired liver function is not equal to liver damage but rather a diverted synthesis of proteins (i.e., APP) during inflammation. This effect in the present study, coupled with the blood albumin and ECM data, corroborates a benign inflammatory condition produced by YC supplementation.

Our results agree with others, where transition dairy cows presented higher concentrations of GGT at 30 DIM than earlier in lactation [32,33]. Furthermore, few studies have reported the effects of yeast supplementation on GGT levels in dairy cows [34,35]. Yeast cell wall supplementation did not affect GGT concentrations in lactating dairy cows [35], but the levels were higher than those observed in our study. In contrast, live yeast supplementation did not affect the GGT levels in early lactating dairy cows; however, the concentration was lower than the observed in our study [34]. Based on the GGT concentrations and other blood biomarkers, we theorize that YC-fed cows were in a mild inflammatory condition, which was not enough to cause negative effects on liver activity.

### 4.3. Gene Expression

Yeast culture compounds, including vitamins, polyphenols, and fermentation end products, can mediate interactions between different immune cell populations, including natural killer cells and B lymphocytes [10]. In addition, Jensen et al. [10] indicated that the mode of action of yeast culture remains unknown; however, this could be partially supported by an initial cellular interaction in gut mucosal tissue, resulting in changes in blood metabolites and cytokine profile. In our study, we observed that yeast culture altered blood biomarkers related to inflammation and liver function while also modifying PMNL gene expression, confirming a cellular interaction between bioactive compounds in yeast culture and the local gut immune system leading to a systemic effect.

The effects of yeast culture have been reported on genes related to inflammation across different species [11,36,37]. Zanello et al. [37] showed that *Saccharomyces cerevisiae* (strain CNCM I-3856) exerted a contrasting downregulation (e.g., *IL6* and *IL8*) and upregulation (e.g., *CCL25*) of genes related to inflammation in porcine intestinal epithelial cells. In weaning pigs supplemented with *S. cerevisiae* fermentation products, Weedman et al. [38] reported an upregulation of *TLR2* and *TLR4* in mesenteric lymph nodes on 1 d after weaning, suggesting that yeast culture had primed immune cells for the changes that occurred during the weaning period. In PMNLs collected from uterine swabs from transition dairy cows, Yuan et al. [11] observed a lack of response to yeast culture on classic molecular markers of inflammation such as *IL6* and *IL8*. In whole blood mRNA expression from newly weaned beef steers, supplementation with a *Saccharomyces-cerevisiae*-based product increased the expression of genes responsible for promoting the animal’s immune response, including *TLR2*, *TNF*, *IL8*, and *ICAM1*, among others [36].

To the best of our knowledge, there are no published data evaluating gene expression in PMNLs from transition dairy cows in response to yeast culture supplementation. In the current study, we observed an upregulation of inflammation-related genes, namely *NFKB1*, *TNFA, TRAF6,* and *IL10,* in YC-fed cows. During pro-inflammatory conditions, TNF Receptor Associated Factor 6 (TRAF6) is upregulated, which encodes a protein that functions as a signal transducer in the nuclear factor κ B subunit 1 (NF-κB1) pathway. The activation of the NF-κB1 pathway is then involved in the release of cytokines, such as *TNFA* and *IL10*, as well as several other proteins related to inflammation [39,40]. As previously discussed, the upregulation of *TRAF6* and *NFKB1* in cows supplemented with YC can be associated with alterations in blood biomarkers related to inflammation and liver function (i.e., reduced albumin and increased GGT). The effects in blood metabolites indicating an inflammatory condition in YC cows could be reflected in the expression of *TRAF6* and *NFKB1* and, consequently, activation of other cytokines.

Interleukin 10 is a cytokine produced primarily by macrophages commonly associated with the resolution of inflammation [41,42]. Interleukin 10 expression has been reported in transition dairy cows in different conditions [43,44]. When fed a high-energy diet during prepartum, transition dairy cows increased the PMNL expression of anti-inflammatory *IL10* at −14 and 7 DIM [43]. According to Moore et al. [41], IL10 may inhibit pro-inflammatory cytokine synthesis, limiting the duration and pathology of the inflammatory response. In our study, YC cows showed an upregulation of pro-inflammatory cytokines (e.g., *NFKB1*, *TRAF6*, and *TNFA)* coupled with the upregulation of *IL10*. Therefore, this concomitant upregulation of *IL10* and pro-inflammatory genes by yeast culture supplementation during the transition period suggests an attenuated inflammatory response, which might be beneficial considering cows experience immune dysfunction during this period. 

In terms of cell receptors and signaling, we observed an upregulation of *SELL*, *ZBP1,* and *VCL* in YC cows. The *SELL* gene encodes the L-selectin protein expressed in the cell surface of PMNLs, which mediates its initial attachment to inflammation sites on the endothelium [45], facilitating leukocytes to leave the bloodstream towards the site of inflammation [46]. Meanwhile, the *ZBP1* gene encodes the Z-DNA binding protein 1, which plays a role in the innate immune response by sensing cell death and microbial pathogens, including viruses not recognized by TLR2 or TLR4 [47]. Regarding *VCL*, it encodes a protein in the cell as soluble, cytoplasmic, and membrane-bound, which is involved in cell functions such as adhesion, motility, and spreading [48,49]. Moreover, *VCL* has been associated with immune function, reducing the susceptibility to virus infections [50,51]. Overall, those results might indicate that peripartal YC supplementation affects neutrophil activation and function.

During early postpartum, high-producing dairy cows are particularly susceptible to the occurrence of oxidative stress, resulting in negative outcomes in terms of productivity and overall health. Oxidative stress is developed due to an increased plasma NEFA, augmented metabolic demand, and inflammatory-like conditions following calving [52,53]. Yeast-culture-fed cows presented a trend for upregulation of the transcription factor nuclear factor erythroid 2-related factor 2 (*NRF2*). *NRF2* is an important regulator of endogenous antioxidant systems. *NRF2* activation promotes the transcription of genes associated with antioxidant defense [54], including *SOD1,* which encodes the superoxide dismutase 1 enzyme [55]. The trends for a concomitant upregulation of *NRF2* and *SOD1* (Table 2) seem to follow the NRF2 mechanism to activate endogenous antioxidant defense systems. 

Parity effects on gene expression regulation in PMNLs have seldom been reported in dairy cows. For instance, Scholte et al. [56] observed an upregulation of genes related to inflammation (i.e., *IL1B*, *IL6*, and *TNFA*) and cell adhesion (*ICAM*) in primiparous cows in comparison to multiparous during the transition period. This suggests that younger animals have a more active immune response or better immunocompetence than older cows, where milk production might restrict nutrients to sustain a viable immune response. The latter is commonly ascribed to a period of negative energy balance (NEB), and older cows likely have a more pronounced NEB in order to maintain higher milk production. In our companion paper [14], the parity effect was not significant (*p* = 0.07) for energy balance as %, but this effect resulted in a trend (*p* = 0.10) for lower EB postpartum in third lactation cows compared to second (88.5% vs. 83%). This effect could potentially explain the Parity × Trt interactions observed in *IL10*, *ZBP1*, *VCL*, and *SOD1*, where PMNLs from second lactation YC cows had an upregulation of these genes in comparison to control. The authors speculate that second lactation YC-fed cows might have responded better to yeast culture supplementation due to a potentially lesser decline in NEB postpartum in comparison to third lactation cows, consequently improving the resolution of inflammation (*IL10*), pathogen detection (*ZBP1*), cell adhesion (*VCL*), and antioxidant systems (*SOD1*). 

Myeloperoxidase is a peroxidase enzyme released during neutrophil activation, which catalyzes the formation of hypochlorous acid, a potent oxidant with bactericidal activity [44]. Although important for host defense, when excessive or misplaced, hypochlorous acid can cause tissue damage and further contribute to oxidative stress [57]. Moreover, MPO activity has been shown to be decreased in the presence of IL-10 [58,59]. Those findings are in line with the results of the present study, where YC-fed cows presented an increase in *IL10* mRNA expression in contrast with the trend for lower *MPO* expression. *Saccharomyces-cerevisiae*-based culture products have been shown to stimulate intracellular antioxidant capacity in vitro [10]. In contrast, *S. cerevisiae* in vivo supplementation as a fermentation product in periparturient dairy cows [12] resulted in mild or no antioxidant effects. 

Based on gene expression results, it is plausible that yeast culture supplementation elicits immune and antioxidant responses in transition dairy cows. This is partially due to PMNLs in YC cows being more stimulated than control cows, suggesting that YC-fed cows were exposed to an inflammatory stimulus and potentially triggered a cellular programming for antioxidant systems. We can speculate that bioactive compounds in yeast culture, such as polyphenols and vitamins, can bypass ruminal fermentation and promote PMNL gene expression alterations. The exact mechanism these bioactive compounds in yeast culture may use to escape ruminal fermentation and interact with immune cells is still inconclusive. We could also hypothesize that bioactive compounds might interact with membrane receptors in the ruminal epithelium, promoting the recruitment of immune cells and, consequently, an immune response.

## 5. Conclusions

Supplementation of yeast culture derived from *Saccharomyces cerevisiae* has been examined for many years to improve performance and health in dairy cows. In transition dairy cows, it is well known that the immune system is compromised, and this type of supplementation can be helpful during this challenging period. Based on blood biomarkers and PMNL mRNA expression, the present study showed that yeast culture could promote a more active inflammatory process with signs of a resolution of the inflammation in transition dairy cows. The immunomodulatory effect of *Saccharomyces cerevisiae* might be associated with a natural response to yeast culture bioactive compounds that can activate the immune system. In a companion paper, we reported that peripartal yeast culture supplementation elicits positive responses in postpartal performance, such as milk yield, SCC, and ECM. Therefore, these results confirm that yeast culture could stimulate the immune system in transition dairy cows while promoting improvements in postpartal performance. 

## Figures and Tables

**Table 1:** Blood biomarkers related to liver function and inflammation for dairy cows fed a basal diet (Control) or a basal diet supplemented with a yeast culture fermentation product (YC) from −31 ± 6 d relative to calving through 50 DIM.

Parameter ^1^	Treatment			*p-Value* ^3^			
	Control	YC	SEM ^2^	Trt	Parity	Time	Trt × T
***Liver function***							
Total bilirubin, µmol/L ^4^	1.65	1.88	0.17	0.42	---	<0.01	0.77
GOT, U/L	95.0	95.0	3.18	0.99	---	<0.01	0.90
GGT, U/L ^4^	18.0	19.9	0.04	0.03	0.03	<0.01	0.03
Cholesterol, mmol/L	3.70	3.49	0.10	0.20	---	<0.01	0.96
PON, U/mL	106.2	97.9	3.50	0.12	0.03	<0.01	0.16
***Inflammation***							
Albumin, g/L	36.0	34.1	0.31	<0.01	0.15	<0.01	0.53
Ceruloplasmin, µmol/L	2.43	2.46	0.06	0.72	---	0.03	0.15
Haptoglobin, g/L ^4^	0.27	0.33	0.15	0.25	---	0.09	0.59
IL-1β, pg/mL ^4^	33.8	31.5	0.18	0.70	0.09	<0.01	0.23

^1^ GOT = glutamic-oxaloacetic transaminase; GGT = gamma-glutamyl transferase; IL-1β = interleukin 1 beta; PON = paraoxonase; ^2^ Largest standard error of the mean is shown; ^3^ Parity × Trt interaction was significant (*p* = 0.05) for GOT. Parity × Time interaction was significant (*p* ≤ 0.05) for PON and GGT. Parity × Trt × Time interaction was significant (*p* = 0.05) for GOT. Trt × T = Interaction of treatment and days in milk; ^4^ Data were log-transformed before statistics. The standard errors of the means associated with log-transformed data are in log scale.

**Table 2:** Polymorphonuclear leukocyte gene expression of target genes related to inflammation, cell receptors and signaling, and oxidative stress in dairy cows fed a basal diet (Control) or a basal diet supplemented with a yeast culture fermentation product (YC) from −31 ± 6 d relative to calving through 50 DIM.

Gene ^1^	Treatment ^2^		SEM ^3^	*p-Value* ^4^			
	Control	YC		Trt	Parity	Time	Trt × T
***Inflammation***							
*TNFA*	0.40	1.23	0.25	0.04	---	<0.01	0.21
*NFKB1*	−0.02	0.23	0.07	0.04	---	0.33	0.42
*IL1B*	0.13	−0.04	0.28	0.68	---	0.94	0.72
*IL10*	0.001	1.36	0.27	0.04	---	<0.01	0.21
*TRAF6*	0.04	0.45	0.15	0.07	---	0.08	0.16
*MYD88*	−0.10	0.12	0.14	0.30	---	0.75	0.23
***Cell receptors and signaling***							
*TLR2*	0.14	0.41	0.12	0.11	0.05	0.04	0.32
*TLR4*	−0.08	0.15	0.17	0.33	---	0.83	0.45
*SELL*	−0.20	0.24	0.10	0.01	0.12	0.83	0.02
*IRAK1*	0.10	0.26	0.12	0.37	---	0.24	0.73
*ZBP1*	−0.07	0.89	0.17	<0.01	0.02	0.15	0.02
*ITGAM*	−0.83	−0.68	0.19	0.56	---	<0.01	0.91
*TLN1*	−0.12	0.009	0.07	0.22	---	0.15	0.52
*VCL*	−0.63	−0.32	0.12	0.08	---	<0.01	0.41
***Oxidative stress***							
*NRF2*	0.12	0.67	0.23	0.09	0.06	0.06	0.27
*SOD1*	−0.19	0.52	0.27	0.07	---	0.67	0.67
*SOD2*	0.11	0.17	0.29	0.88	0.07	0.27	0.81
*MPO*	−2.00	−3.27	0.52	0.10	---	<0.01	0.23

^1^*TNFA* = tumor necrosis factor alpha; *NFKB1* = nuclear factor kappa B subunit 1; *IL1B* = interleukin 1 beta; *IL10* = interleukin 10; *TRAF6* = TNF receptor associated factor 6; *MYD88* = myeloid differentiation primary response 88; *TLR2* = toll-like receptor 2; *TLR4* = toll-like receptor 4; *SELL* = selectin L; *IRAK1* = interleukin 1 receptor associated kinase 1; *ZBP1* = Z-DNA binding protein 1; *ITGAM* = integrin subunit alpha M; *TLN1* = talin 1; *VCL* = vinculin; *NRF2* = nuclear factor erythroid 2-related factor 2; *SOD1* = superoxide dismutase 1; *SOD2* = superoxide dismutase 2; *MPO* = myeloperoxidase; ^2^ Fold change data were log-transformed before statistics; ^3^ Largest standard error of the mean is shown; ^4^ Parity × Trt interaction was significant (*p* = 0.04) for *IL10*, *ZBP1*, *SOD1*, and *VCL*. Parity × Trt × Time interaction was significant (*p* = 0.02) for ZBP1. Trt × T = interaction of treatment and days in milk.

## Data Availability

Not applicable.

## Supplementary Materials

The following supporting information can be downloaded at: https://doi.org/10.6084/m9.figshare.21625175.v3, Table S1: GenBank accession number, hybridization position, sequence and amplicon size of primers *for Bos taurus* used to analyze gene expression by qPCR; Table S2: Genes selected for transcript profiling in bovine polymorphonuclear leukocytes (PMNL) and their biological functions^1^; Figure S1: Independent analysis of the Parity × treatment effect in polymorphonuclear leukocyte mRNA expression of interleukin 10 (gene symbol IL10; *p* = 0.04), Z-DNA binding protein 1 (gene symbol ZBP1; *p* < 0.01), vinculin (gene symbol VCL; *p* = 0.02), superoxide dismutase 1 (gene symbol SOD1; *p* = 0.05). Difference in mRNA expression between YC cows and control in 2nd lactation cows is denoted by an asterisk (*) for IL10 (*p* = 0.01), ZBP1 (*p* < 0.01), VCL (*p* = 0.01), and SOD1 (*p* = 0.02). Values are least square means; error bars represent standard errors; Figure S2: Percent change in blood albumin and gamma-glutamyl transferase (GGT) relative to − 30 DIM in cows (*n* = 8/treatment) supplemented with a basal diet (Control) or a basal diet plus yeast culture fermentation production (YC) from − 31 ± 6 d relative to parturition through 50 DIM. Mean separations between treatments at a given time point were evaluated when a treatment × time (Trt × T) interaction (*p* ≤ 0.05) was observed, and differences (*) were declared at a Tukey-adjusted *p* ≤ 0.05. Values are means, and SE are represented by vertical bars.
